# Supplementary material for: Spike Formation Is a Turning Point Determining Wheat Root Microbiome Abundance, Structures and Functions
Source: Int J Mol Sci. 2021 Nov 4;22(21):11948. doi: 10.3390/ijms222111948 (PMC8585012; doi:10.3390/ijms222111948)

**Additional file 1**

**Supplemental Table S1.** Primers used for quantitative PCR.

| Primer name | Primer sequence (5'→3') | Amplicon size (bp) | Master mix used for qPCR | Thermal profile of qPCR | Reference |
|---|---|---|---|---|---|
| nosZ-1826Fm | GSC AGR TSK KSA AGT GGA A | 186 | Power SYBR MasterMix | 95°C for 10 min, 40 cycles at 95°C for 15 s, 62°C for 1 m, 95°C for 10s (+0.3) | [1] |
| nosZ-2012R | CGG TCC TTS GAG AAC TT | | | | |
| 16S-331F | TCC TAC GGG AGG CAG CAG T | 195 | FAST SYBR MasterMix | 95°C for 5 min, 40 cycles at 95°C for 5 s, 60°C for 30 s, 95°C for 10s (+0.3) | [2, 3] |
| 16S-518R | ATT ACC GCG GCT GCTG G | | | | |
| TEF_F | ACT GTG CAG TAG TAC TTG GTG | 155 | FAST SYBR MasterMix | 95°C for 5 min, 40 cycles at 95°C for 5 s, 60°C for 30 s, 95°C for 10s (+0.3) | [4] |
| TEF_R | AAG CTA GGA GGT ATT GAC AAG | | | | |


1. Usyskin-Tonne A, Hadar Y, Yermiyahu U, Minz D. Elevated $CO_2$ has a significant impact on denitrifying bacterial community in wheat roots. *Soil Biol Biochem* 2020; **142**: 107697.
2. Hunter N, Nadkarni MA, Jacques NA, Martin FE. Determination of bacterial load by real-time PCR using a broad-range (universal) probe and primers set. *Microbiology* 2002; **148**: 257–266.
3. Lopez I, Ruiz-Larrea F, Cocolin L, Orr E, Phister T, Marshall M, et al. Design and evaluation of PCR primers for analysis of bacterial populations in wine by denaturing gradient gel electrophoresis. *Appl Environ Microbiol* 2003; **69**: 6801–6807.
4. Ruppel S, Rühlmann J, Merbach W. Quantification and localization of bacteria in plant tissues using quantitative real-time PCR and online emission fingerprinting. *Plant Soil* 2006; **286**: 21–35.

**Supplemental Table S2.** Root-associated and rhizosphere contigs divided into catalogues

| Niche | Catalogue name | Number of contigs | N50 (bp) |
|---|---|---|---|
| Roots | week 1 | 233,475 | 700 |
| | week 2 | 295,394 | 780 |
| | week 5 | 349,966 | 691 |
| | week 9 | 265,158 | 702 |
| | week 12 | 439,827 | 736 |
| | week 17a | 371,640 | 759 |
| | week 17b | 567,046 | 680 |
| Rhizosphere | week 1 | 470,984 | 701 |
| | week 2 | 485,480 | 706 |
| | week 5 | 557,525 | 726 |
| | week 9 | 458,671 | 722 |
| | week 12a | 266,674 | 694 |
| | week 12b | 294,897 | 692 |
| | week 17a | 274,544 | 689 |
| | week 17b | 264,367 | 685 |

**Supplemental Table S3.** List of normalized gene counts per cell for *Proteobacteria*, *Actinobacteria* and *Bacteroidetes*

| Bacteria phyla | Function group | Function group type | Relative abundance (%) | Number of genes before spike formation | Number of genes after spike formation | Functional genes significaly abundant before or after spike formation | Number of genes per cell |
|---|---|---|---|---|---|---|---|
| Actinobacteria | Amino acid | ABC transporters | 27 | 809 | 715 | before | 30 |
| Actinobacteria | Amino acid | ABC transporters | 37 | 7381 | 12003 | after | 325 |
| Actinobacteria | Amino acid | Biosynthesis | 27 | 3 | 3 | before | 0 |
| Actinobacteria | Amino acid | Biosynthesis | 37 | 5457 | 9522 | after | 258 |
| Actinobacteria | Amino acid | Degradation | 27 | 1532 | 2327 | before | 57 |
| Actinobacteria | Amino acid | Metabolism | 27 | 18848 | 28884 | before | 702 |
| Actinobacteria | Amino acid | Metabolism | 37 | 3400 | 5278 | after | 143 |
| Actinobacteria | Antibiotics synthesis and resistance | ABC transporters | 27 | 56 | 22 | before | 2 |
| Actinobacteria | Antibiotics synthesis and resistance | ABC transporters | 37 | 1068 | 1848 | after | 50 |
| Actinobacteria | Antibiotics synthesis and resistance | Biosynthesis | 37 | 10672 | 19366 | after | 524 |
| Actinobacteria | Antibiotics synthesis and resistance | Resistance | 27 | 4063 | 6187 | before | 151 |
| Actinobacteria | Antibiotics synthesis and resistance | Resistance | 27 | 279 | 557 | before | 10 |
| Actinobacteria | Antibiotics synthesis and resistance | Transporters | 27 | 133 | 236 | before | 5 |
| Actinobacteria | Chemotaxis | | 27 | 3332 | 6834 | before | 124 |
| Actinobacteria | Quorum sensing | | 27 | 16740 | 24655 | before | 624 |
| Actinobacteria | Secretion system | | 27 | 2142 | 3301 | before | 80 |
| Actinobacteria | Biofilm formation | | 27 | 7643 | 12537 | before | 285 |
| Actinobacteria | Carbon | ABC transporters | 27 | 828 | 1429 | before | 31 |
| Actinobacteria | Carbon | ABC transporters | 37 | 27467 | 46799 | after | 1267 |
| Actinobacteria | Carbon | Biosynthesis | 27 | 5617 | 6387 | before | 209 |
| Actinobacteria | Carbon | Biosynthesis | 37 | 6121 | 9364 | after | 253 |
| Actinobacteria | Carbon | Degradation | 37 | 10924 | 17979 | after | 487 |
| Actinobacteria | Carbon | Metabolism | 27 | 2854 | 4229 | before | 106 |
| Actinobacteria | Carbon | Metabolism | 37 | 42138 | 73426 | after | 1988 |
| Actinobacteria | Carbon | Transporters | 27 | 42 | 16 | before | 2 |
| Actinobacteria | Ion | ABC transporters | 27 | 8 | 8 | before | 0 |
| Actinobacteria | Ion | Transporters | 27 | 44 | 28 | before | 2 |
| Actinobacteria | Metal | ABC transporters | 27 | 2561 | 4684 | before | 95 |
| Actinobacteria | Metal | ABC transporters | 37 | 325 | 621 | after | 17 |
| Actinobacteria | Nitrogen | Denitrification | 27 | 501 | 780 | before | 19 |
| Bacteroidetes | Amino acid | ABC transporters | 5 | 29 | 17 | before | 6 |
| Bacteroidetes | Amino acid | ABC transporters | 5 | 8 | 34 | after | 6 |
| Bacteroidetes | Amino acid | Biosynthesis | 5 | 416 | 695 | after | 132 |
| Bacteroidetes | Amino acid | Degradation | 5 | 128 | 156 | before | 24 |
| Bacteroidetes | Amino acid | Metabolism | 5 | 5114 | 6912 | before | 975 |
| Bacteroidetes | Amino acid | Metabolism | 5 | 491 | 604 | after | 115 |
| Bacteroidetes | Antibiotics synthesis and resistance | ABC transporters | 5 | 7 | 5 | before | 1 |
| Bacteroidetes | Antibiotics synthesis and resistance | ABC transporters | 5 | 37 | 59 | after | 11 |
| Bacteroidetes | Antibiotics synthesis and resistance | Biosynthesis | 5 | 1001 | 1298 | after | 247 |
| Bacteroidetes | Antibiotics synthesis and resistance | Resistance | 5 | 2264 | 2751 | before | 453 |
| Bacteroidetes | Antibiotics synthesis and resistance | Transporters | 5 | 251 | 284 | before | 48 |
| Bacteroidetes | Chemotaxis | | 5 | 254 | 480 | before | 48 |
| Bacteroidetes | Quorum sensing | | 5 | 1314 | 1721 | before | 251 |
| Bacteroidetes | Secretion system | | 5 | 74 | 47 | before | 14 |
| Bacteroidetes | Biofilm formation | | 5 | 545 | 793 | before | 104 |
| Bacteroidetes | Carbon | ABC transporters | 5 | 780 | 1003 | before | 149 |
| Bacteroidetes | Carbon | ABC transporters | 5 | 17 | 20 | after | 4 |
| Bacteroidetes | Carbon | Biosynthesis | 5 | 1760 | 1786 | before | 335 |
| Bacteroidetes | Carbon | Biosynthesis | 5 | 822 | 1317 | after | 251 |
| Bacteroidetes | Carbon | Degradation | 5 | 4939 | 7264 | after | 1382 |
| Bacteroidetes | Carbon | Metabolism | 5 | 18 | 20 | before | 3 |
| Bacteroidetes | Carbon | Metabolism | 5 | 8230 | 11833 | after | 2251 |
| Bacteroidetes | Ion | ABC transporters | 5 | 78 | 51 | before | 15 |
| Bacteroidetes | Ion | Transporters | 5 | 94 | 123 | before | 18 |
| Bacteroidetes | Ion | Transporters | 5 | 5 | 2 | before | 1 |
| Bacteroidetes | Metal | ABC transporters | 5 | 73 | 62 | before | 14 |
| Bacteroidetes | Nitrogen | Denitrification | 5 | 41 | 25 | before | 8 |
| Proteobacteria | Amino acid | ABC transporters | 62 | 27986 | 23593 | before | 450 |
| Proteobacteria | Amino acid | ABC transporters | 52 | 4231 | 4492 | after | 87 |
| Proteobacteria | Amino acid | Biosynthesis | 62 | 7 | 17 | before | 0 |
| Proteobacteria | Amino acid | Biosynthesis | 52 | 3341 | 4203 | after | 82 |
| Proteobacteria | Amino acid | Degradation | 62 | 7584 | 6383 | before | 122 |
| Proteobacteria | Amino acid | Metabolism | 62 | 78019 | 70650 | before | 1253 |
| Proteobacteria | Amino acid | Metabolism | 52 | 6463 | 7226 | after | 140 |
| Proteobacteria | Antibiotics synthesis and resistance | ABC transporters | 62 | 11196 | 9291 | before | 180 |
| Proteobacteria | Antibiotics synthesis and resistance | ABC transporters | 52 | 51 | 46 | after | 1 |
| Proteobacteria | Antibiotics synthesis and resistance | Biosynthesis | 52 | 6975 | 6541 | after | 127 |
| Proteobacteria | Antibiotics synthesis and resistance | Resistance | 62 | 36956 | 36280 | before | 594 |
| Proteobacteria | Antibiotics synthesis and resistance | Resistance | 62 | 17899 | 15325 | before | 289 |
| Proteobacteria | Antibiotics synthesis and resistance | Transporters | 62 | 36841 | 30887 | before | 592 |
| Proteobacteria | Chemotaxis | | 62 | 47886 | 39568 | before | 769 |
| Proteobacteria | Quorum sensing | | 62 | 53297 | 52493 | before | 856 |
| Proteobacteria | Secretion system | | 62 | 86817 | 67617 | before | 1395 |
| Proteobacteria | Biofilm formation | | 62 | 41563 | 37687 | before | 668 |
| Proteobacteria | Carbon | ABC transporters | 62 | 30072 | 25734 | before | 483 |
| Proteobacteria | Carbon | ABC transporters | 52 | 9950 | 10446 | after | 203 |
| Proteobacteria | Carbon | Biosynthesis | 62 | 15081 | 13209 | before | 242 |
| Proteobacteria | Carbon | Biosynthesis | 52 | 12626 | 13861 | after | 269 |
| Proteobacteria | Carbon | Degradation | 52 | 7271 | 8049 | after | 156 |
| Proteobacteria | Carbon | Metabolism | 62 | 13902 | 12629 | before | 223 |
| Proteobacteria | Carbon | Metabolism | 52 | 55133 | 53344 | after | 1035 |
| Proteobacteria | Carbon | Transporters | 62 | 2101 | 1498 | before | 34 |
| Proteobacteria | Ion | ABC transporters | 62 | 4067 | 3169 | before | 65 |
| Proteobacteria | Ion | Transporters | 62 | 290 | 89 | before | 5 |
| Proteobacteria | Ion | Transporters | 62 | 8365 | 6147 | before | 134 |
| Proteobacteria | Metal | ABC transporters | 62 | 25229 | 22403 | before | 405 |
| Proteobacteria | Nitrogen | Denitrification | 62 | 7715 | 5879 | before | 124 |

**Supplemental Figure S1.** Weather conditions during wheat growth

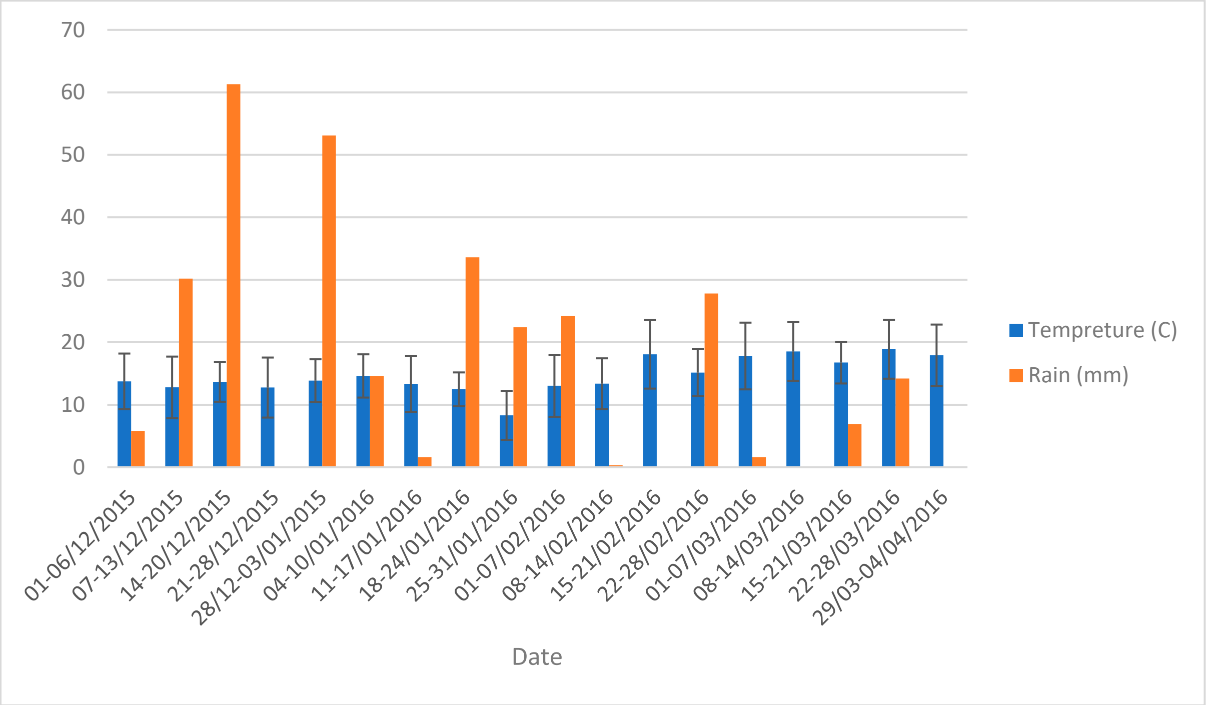


Error bars indicate average temperature during a day (maximum) and average temperature during a night (minimum).

**Supplemental Figure S2.** Green (A) and yellow (B) spikes at the final stages of wheat growth (17 week)

A)

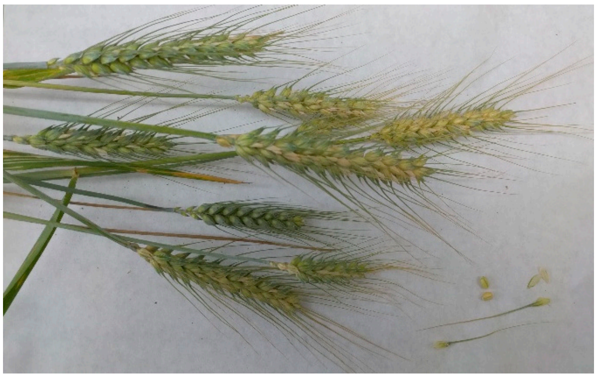

B)

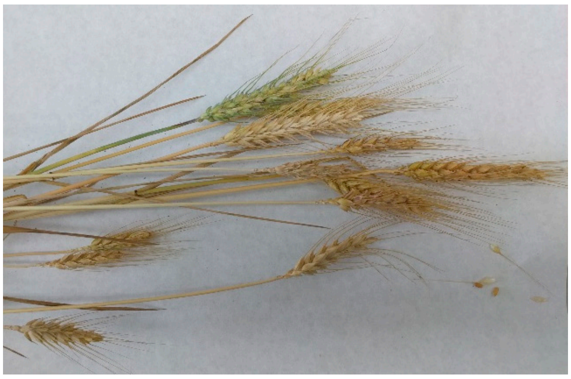

Supplement: Supplementary file 1 [file ijms-22-11948-s001.zip › ijms-1432936-supplementary.pdf]
